# Novel Application of a New Lateral System for Adjacent-level Revision Surgery: A Case Report

**DOI:** 10.7759/cureus.5092

**Published:** 2019-07-07

**Authors:** John Paul G Kolcun, G Damian Brusko, Michael Y Wang

**Affiliations:** 1 Neurosurgery, University of Miami Miller School of Medicine, Miami, USA

**Keywords:** lateral lumbar interbody fusion, minimally invasive spine surgery (miss), duo system, spine, revision fusion, adjacent-segment disease

## Abstract

In recent years, lateral lumbar interbody fusion (LLIF) has grown in popularity as a minimally invasive spine surgery (MISS) approach that can be offered to patients with prior surgeries from a posterior approach. In this report, we present a patient with a focal disease and a history of multiple posterior lumbar surgeries who underwent LLIF with a novel application of the Duo^TM^ system (Spineology Inc., MN, USA) adjacent to prior surgical levels and without posterior instrumentation. At one year postoperatively, she continued to have no back pain or complaints relating to her lumbar pathology. The case demonstrates the novel use of a new MISS LLIF system that requires minimal exposure as compared to current LLIF systems to treat a patient with adjacent segment disease and progressive symptoms following multiple posterior decompressive surgeries.

## Introduction

Modern techniques in minimally invasive spine surgery (MISS) have found increasing popularity among patients who fear painful operations and prolonged hospitalizations. For selected patients with appropriate pathology, MISS can offer an effective means of alleviating symptomatology with a mild hospital course and superior cosmetic outcomes.

MISS approaches can be especially applicable in reoperations, where traditional surgical corridors may be less advantageous due to anatomical distortion and scar tissue formation [1]. In recent years, lateral lumbar interbody fusion (LLIF) has grown in popularity as a MISS approach and can be offered to patients with prior surgeries from a posterior approach.

In this report, we present a patient with a focal disease and a history of multiple posterior lumbar surgeries who underwent LLIF with a novel application of the Duo^TM^ system (Spineology Inc., MN, USA) adjacent to prior surgical levels and without posterior instrumentation.

## Case presentation

Patient presentation

The patient is a 56-year-old woman with a complicated history of back pain. She was involved in a motor vehicle collision in 2005, 13 years before our first encounter in 2018. She had three prior decompressive lumbar surgeries at an outside hospital in 2007, 2008, and 2014, respectively. These gave her relief initially, but the back pain returned in 2016, possibly related to a fall onto her left shoulder.

She had attempted physical therapy, medical management, including opiates and muscle relaxants, and multiple lumbar injections, which provided only temporary relief. Her medical history was significant for anxiety/depression, exacerbated by her chronic pain. She denied constitutional symptoms such as fever, weight loss, and night sweats.

She reported aching/stabbing pain in the lower back, radiating to the right thigh, from the hip to the groin and extending to the knee but not involving the lower leg. She had no weakness, imbalance, or gait disturbance.

**Figure 1:**
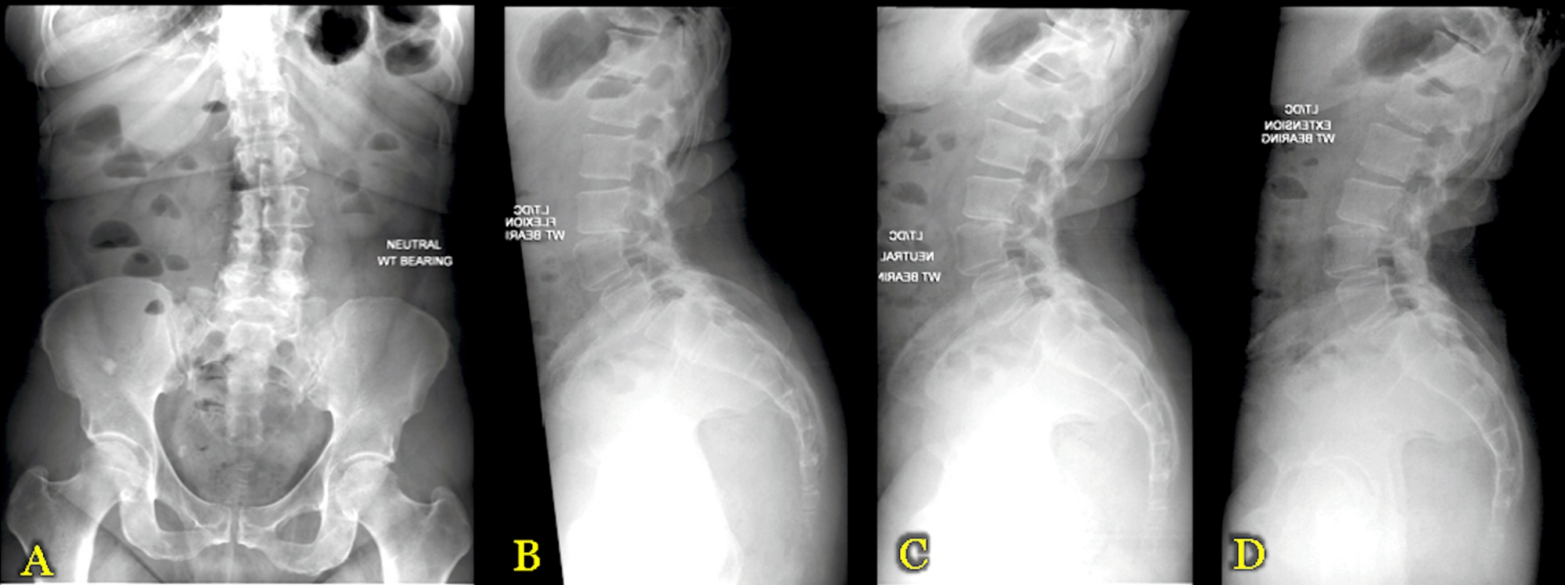
Preoperative X-Ray (XR) Imaging Panel A demonstrates 36" standing anteroposterior (AP) scoliosis XR images. Panel B demonstrates 36" standing lateral scoliosis XR images. Panels C and D demonstrate flexion and extension XR images, respectively.

Imaging studies showed diffuse degenerative changes of the lumbar spine. X-rays demonstrated focal scoliosis with right concavity at the L3-L4 level (Figure 1A) and grade I anterolisthesis of L3-L4 with slight mobility of flexion-extension (Figures 1B-1D).

**Figure 2:**
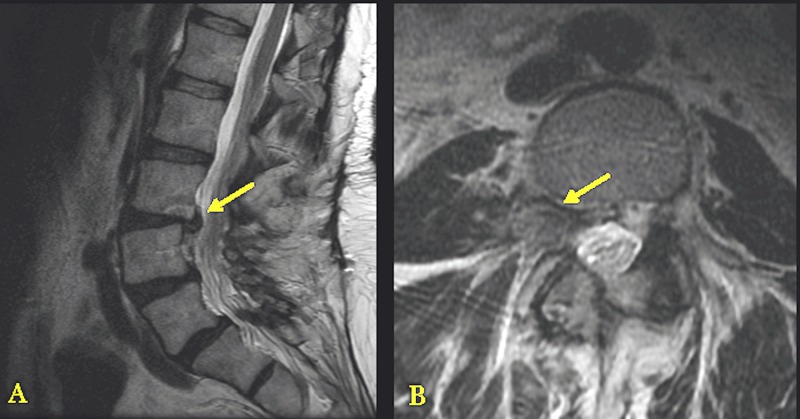
Preoperative Magnetic Resonance Imaging (MRI) Panel A demonstrates lateral MRI with the arrow pointing to the disc herniation at L3-L4. Panel B demonstrates an axial MRI with the arrow pointing to the disc herniation with nerve root impingement at L3-L4.

Magnetic resonance imaging (MRI) was significant for large disc herniation at the L3-L4 level, impinging on the right exiting L3 nerve root (Figure 2). Given the imaging findings consistent with the patient's history of low back pain and radiculopathy, lateral lumbar interbody fusion was recommended to provide indirect decompression of the right L3 nerve root while maintaining spinal stability.

Operative report

The patient was intubated and paralyzed and then placed in the left lateral decubitus position. The right flank was prepped and draped in the usual sterile fashion. After fluoroscopic localization, an incision was made directly over the L3-L4 space. A plane was developed by blunt dissection through the external and internal oblique muscles, followed by the transversalis muscle. A secondary incision of the flank was made to access the retroperitoneal space, in order to facilitate our dissection to the psoas major.

**Figure 3:**
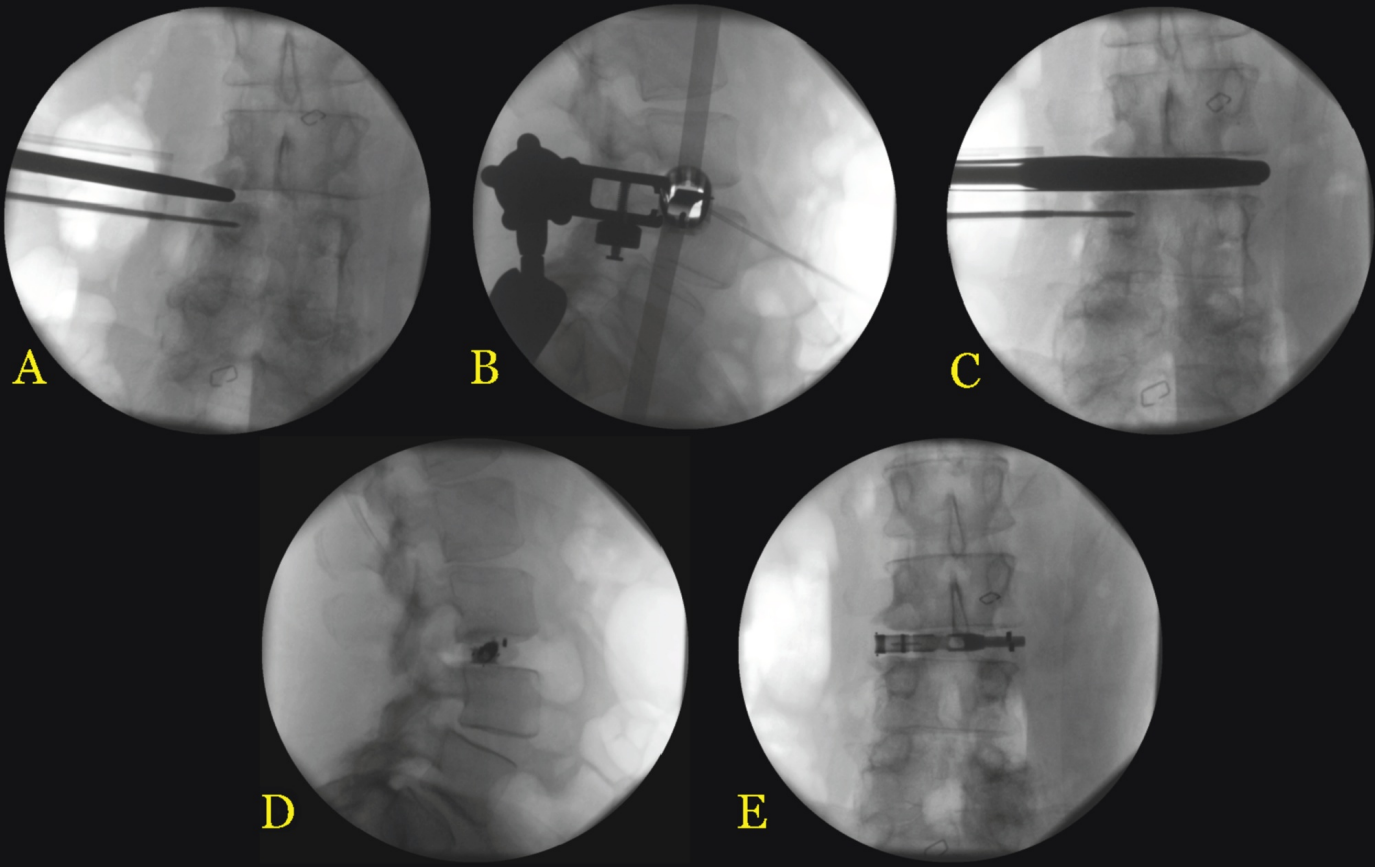
Intraoperative Fluoroscopy Images Panels A and B demonstrate anteroposterior (AP) and lateral images, respectively, of the placement of the tubular retractor system at L3-L4. Panel C demonstrates the opening up of the L3-L4 disc space with a trial spacer. Panels D and E demonstrate the final lateral and AP images, respectively, of the Duo^TM^ implant placed at L3-L4. DuoTM system (Spineology Inc., MN, USA)

Using electrostimulation to obtain a safe trajectory among nearby nerve roots, a guidewire was inserted through the psoas muscle into the target disc space. A series of dilators were used to develop a corridor along the wire, whereby a tubular retractor was introduced at the target L3-L4 disc space and anchored to the L4 vertebra with a pin (Figures 3A-3B). A series of trials and elevators were used to open the disc space (Figure 3C). Discectomy and endplate preparation were performed with micro-instruments specialized for tubular access, including angled curettes and rakes to reach those portions of the disc/endplate anterior and posterior to the tubular channel. Adequate discectomy was confirmed under fluoroscopy by inflating a balloon with radiopaque contrast material within the interbody space. Following endplate preparation, an 8 mm high x 50 mm wide Duo^TM^ implant was placed into the interbody space with extra-small bone morphogenetic protein (BMP) sponges anteriorly and posteriorly. The mesh portion of the graft was filled with a morselized bone allograft until adequate interbody height restoration and indirect decompression of the right L3 nerve root was achieved and the device was seated securely in the disc space. The mesh cage was crimped shut, containing the bone allograft. The stability of the graft seating suggested good ligamentous strength and mechanical stability across this disc level, obviating the need for supporting posterior instrumentation (Figures 3D-3E).

Retractors were removed under direct visualization with flexible fiber-optic lighting, taking care to inspect for vascular injury or hollow viscous bowel injury. Incisions were closed in layers with resorbable sutures, taking care to prevent hernia formation. The patient was flipped supine and extubated at neurologic baseline, with no change on electrophysiologic monitoring.

Postoperative course

**Figure 4:**
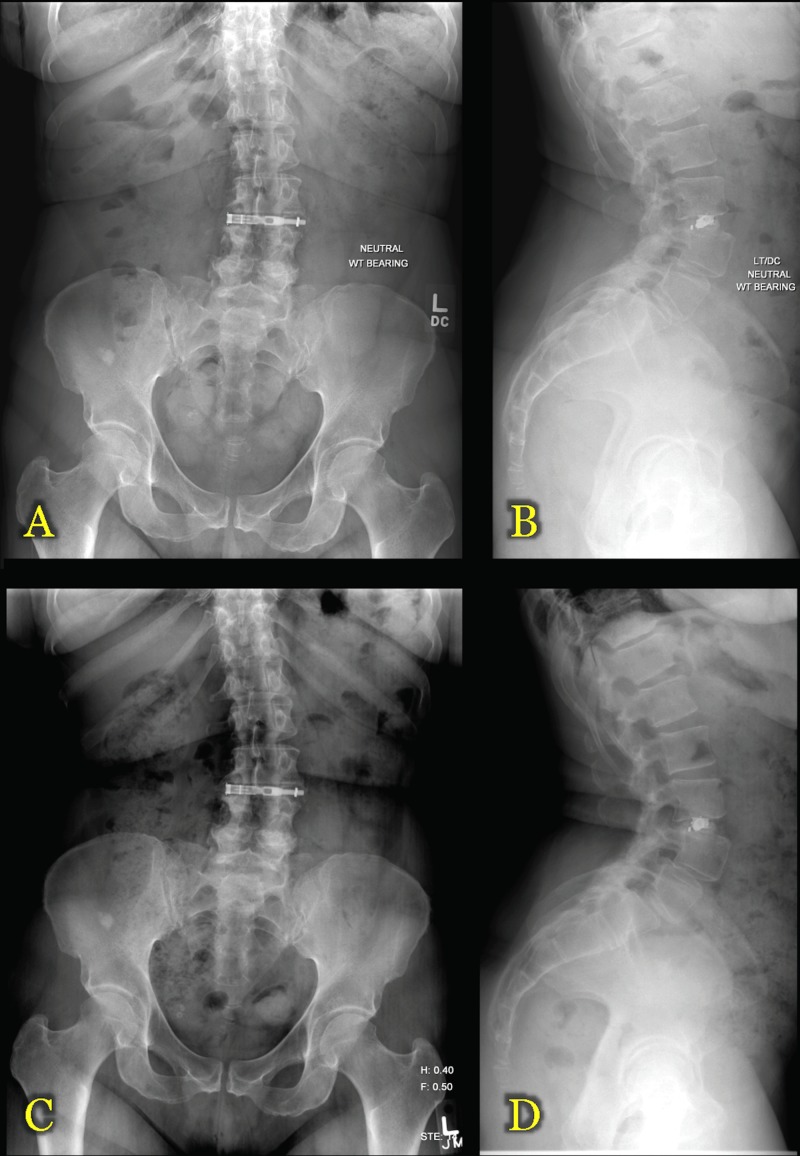
Postoperative X-Ray (XR) Imaging Panels A and B demonstrate 36" standing scoliosis anteroposterior (AP) and lateral XR images, respectively, two months postoperatively. Panels C and D demonstrate 36" standing scoliosis anteroposterior (AP) and lateral XR images, respectively, six months postoperatively.

The patient had an uneventful hospital stay and was discharged home in good condition on postoperative day two. At the two-week postoperative visit, she reported complete resolution of her lower extremity pain and paresthesias with some residual low back pain for which she was taking diazepam 5 mg and oxycodone-acetaminophen 10-325 mg tablets as needed. She was seen in the clinic two months after surgery, reporting near-complete relief of her back pain and only occasional use of analgesics. Imaging at that time (Figures 4A-4B) and six months postoperatively (Figures 4C-4D) demonstrated early signs of fusion through the interbody space and no signs of cage displacement or pseudarthrosis.

**Figure 5:**
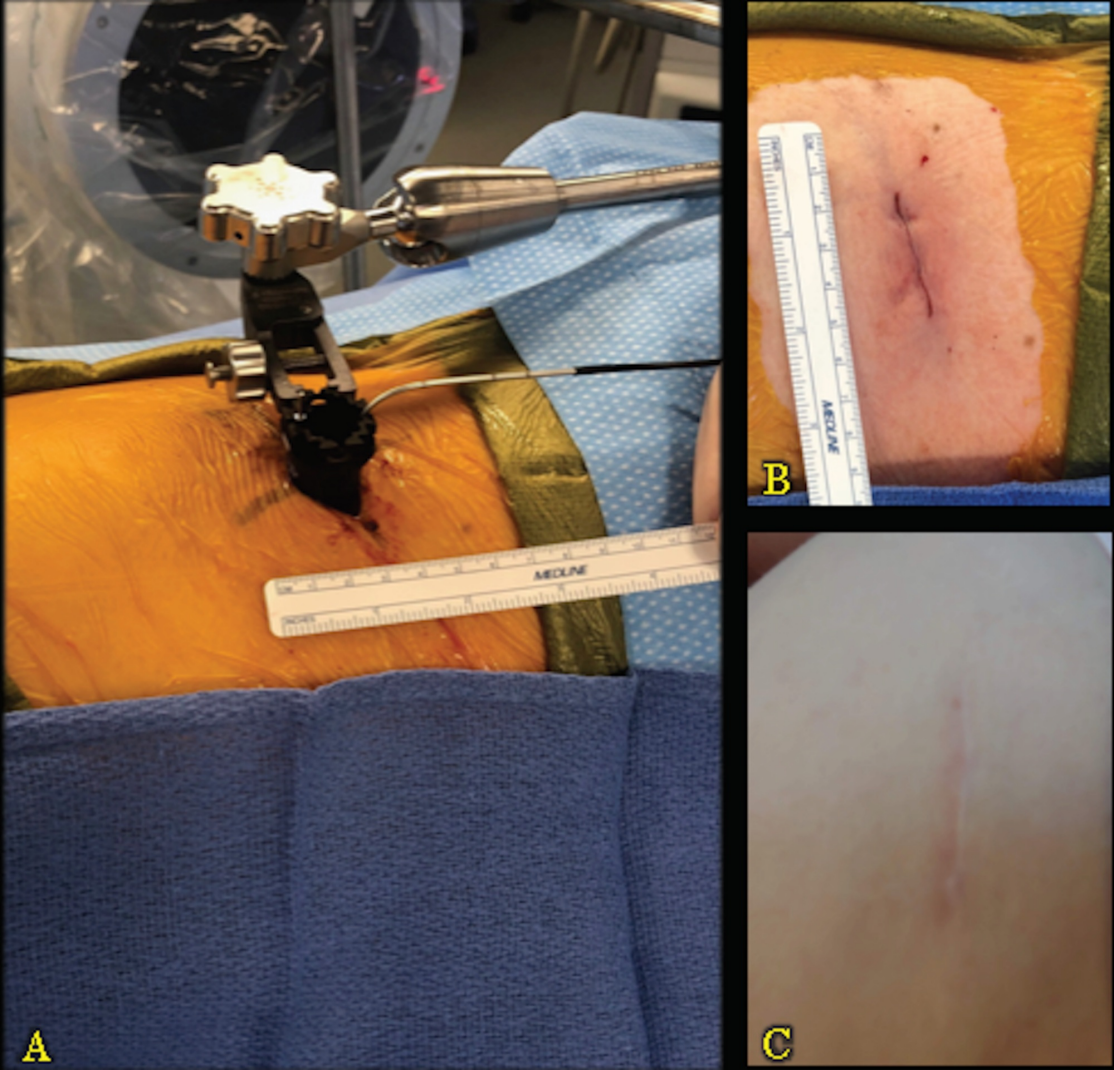
Intraoperative Exposure and Lateral Scar Panel A demonstrates the intraoperative exposure required for docking. Panel B demonstrates the postoperative closure. Panel C demonstrates the patient’s lateral scar one year after surgery.

She was last seen in our clinic one year after her surgery. At that time, she continued to have no back pain or complaints relating to her lumbar pathology. Figure 5C demonstrates the patient’s lateral scar at one year after surgery, in direct comparison with the intraoperative exposure required for docking (Figure 5A) and postoperative closure (Figure 5B).

## Discussion

This case demonstrates a novel application of a new device for LLIF that requires minimal exposure as compared to the common LLIF systems currently in use. After progressive symptoms with local deformity following three previous posterior decompressive surgeries, our patient now has excellent, durable clinical results at one year postoperatively, achieved with a MISS fusion procedure that did not require posterior instrumentation.

The Duo^TM^ interbody device consists of a titanium rod core with two poly-ethyl-ether-ketone spacers on each end, flanking a central expansile mesh containment device. The mesh portion is filled with bone allograft once introduced within the interbody space, allowing for (i) insertion through a narrower channel than with conventional non-expansile or purely rigid interbody devices, (ii) mechanical stability through increased graft footprint after mesh expansion, and (iii) effective indirect decompression through increased interbody height.

As with any interbody fusion, graft subsidence is an important consideration for long-term treatment efficacy through indirect decompression. A recent meta-analysis identified graft subsidence in up to 10% of patients who underwent LLIF, with a height loss of approximately 10% at one year postoperatively [2]. Biomechanical analysis, however, has suggested that LLIF grafts may be less prone to subsidence as compared to posterior or transforaminal LIF grafts due to their greater width and increased footprint in the interbody space [3]. The conforming nature of the expansile mesh portion of the Duo^TM^ graft and the resultant wider graft footprint should, therefore, provide further protection from subsidence in our patient, who did, in fact, have durable symptom relief at one-year postoperatively.

Complications following LLIF can include leg weakness or sensory change, flank bulging, or pseudohernia formation, and, most troublingly, frank injury to the femoral nerve. Our institutional complication rate in the preceding decade has been examined iteratively with the adoption of new lateral approaches, including more traditional percutaneous methods and newer mini-open techniques, and has remained acceptably low [4-5]. We believe that the minimal open access and psoas dissection required with this system, while still achieving direct visualization of the surgical corridor and surrounding structures, should further mitigate the risk of neurovascular injury during the lateral approach.

## Conclusions

The case demonstrates the novel use of a new MISS LLIF system to treat a patient with adjacent segment disease and progressive symptoms following multiple posterior decompressive surgeries. The patient remains symptom-free at one year postoperatively, with no signs of symptom recurrence or mechanical instability.
